# Activation of Cholinergic Anti-Inflammatory Pathway Ameliorates Cerebral and Cardiac Dysfunction After Intracerebral Hemorrhage Through Autophagy

**DOI:** 10.3389/fimmu.2022.870174

**Published:** 2022-06-23

**Authors:** Yue Su, Wei Zhang, Ruoxi Zhang, Quan Yuan, Ruixia Wu, Xiaoxuan Liu, Jimusi Wuri, Ran Li, Tao Yan

**Affiliations:** Department of Neurology, Tianjin Medical University General Hospital, Tianjin Neurological Institute, Key Laboratory of Post-Neurotrauma, Neurorepair, and Regeneration in Central Nervous System, Ministry of Education and Tianjin City, Tianjin, China

**Keywords:** intracerebral hemorrhage, α7nAChR, autophagy, inflammation, macrophage

## Abstract

**Background:**

Intracerebral hemorrhage (ICH) is the devastating subtype of stroke with cardiovascular complications, resulting in high rates of mortality and morbidity with the release of inflammatory factors. Previous studies have demonstrated that activation of α7nAChR can reduce immune and inflammation-related diseases by triggering the cholinergic anti-inflammatory pathway (CAIP). α7nAChR mediates protection from nervous system inflammation through AMPK-mTOR-p70S6K-associated autophagy. Therefore, the purpose of this study is to explore whether the activation of α7nAChR improves cerebral and cardiac dysfunction after ICH through autophagy.

**Methods:**

Male C57BL/6 mice were randomly divided into five groups **(1)**: Control + saline **(2)**, ICH+ saline **(3)**, ICH + PNU-282987 **(4)**, ICH+ PNU-282987 + MLA **(5)**, ICH + PNU-282987 + 3-MA. The neurological function was evaluated at multiple time points. Brain water content was measured at 3 days after ICH to assess the severity of brain edema. PCR, immunofluorescence staining, and Western Blot were performed at 7 days after ICH to detect inflammation and autophagy. Picro-Sirius Red staining was measured at 30 days after ICH to evaluate myocardial fibrosis, echocardiography was performed at 3 and 30 days to measure cardiac function.

**Results:**

Our results indicated that the PNU-282987 reduced inflammatory factors (MCP-1, IL-1β, MMP-9, TNF-α, HMGB1, TLR2), promoted the polarization of macrophage/microglia into anti-inflammatory subtypes(CD206), repaired blood-brain barrier injury (ZO-1, Claudin-5, Occludin), alleviated acute brain edema and then recovered neurological dysfunction. Echocardiography and PSR indicated that activation of α7nAChR ameliorated cardiac dysfunction. Western Blot showed that activation of α7nAChR increased autophagy protein (LC3, Beclin) and decreased P62. It demonstrated that the activation of α7nAChR promotes autophagy and then recovers brain and heart function after ICH.

**Conclusions:**

In conclusion, PNU-282987 promoted the cerebral and cardiac functional outcomes after ICH in mice through activated α7nAChR, which may be attributable to promoting autophagy and then reducing inflammatory reactions after ICH.

## Introduction

Intracerebral hemorrhage (ICH), accounts for 10–15% of strokes (1), remains the leading cause of morbidity and mortality and is associated with severe long-term disability. Brain injury after ICH includes the consequences of primary physical trauma and enlarged hematoma, secondary brain injury (SBI) such as cerebral edema, blood-brain barrier disruption, neuronal death and inflammation may also occur (2), it is characterized by activation of microglia, induction of pro-inflammatory factors and migration of peripheral inflammatory cells into the central nervous system (CNS) (3–5). Our previous research in mice has shown that even with the lack of primary heart disease or risk factors such as hypertension, old age, or diabetes, brain injuries including subarachnoid hemorrhage (SAH), ischemic stroke, ICH, or traumatic brain injury (TBI), could cause cardiac pathological remodeling and cardiac functional deficits (6–9). Acetylcholine (ACh) is a small molecule composed of choline and acetic acid. As a neurotransmitter in the central nervous system and peripheral nervous systems, it plays a key role in maintaining brain function. As one of the two dominant classes, nicotinic ACh receptors (nAChRs) are members of the cysteine-loop family of ligand-gated ion channels (10, 11). The α7 receptor is one of the nAChRs which is abundantly expressed in brain, characterized by high calcium permeability and rapid desensitization (12). It is involved in cellular processes such as survival and plasticity together with higher brain functions such as memory and learning (13). In addition, α7nAChR is expressed on a variety of cells, including glial cells, macrophages, endothelial cells, keratinocytes, dendritic cells, and lymphocytes. Other researchers have previously shown that activation of α7nAChR can alleviate multiple types of inflammatory and immune-related disorders by triggering so-called cholinergic anti-inflammatory pathways in neurological disorders (14–16). However, the underlying mechanisms have not been absolutely elucidated.

Autophagy is a lysosome-dependent pathway of cellular self-defense. Some long-lived proteins, misfolded proteins and damaged organellesare degraded and recycled through the way of autophagy (17–19). Autophagy maintains the stability of the intracellular environment and normal cellular functions. However, excessive autophagy can impair the health of cells and eventually lead to autophagic cell death under various stress conditions. Consequently, autophagy at basal level may serve as a “double-edged sword” (20). Hematoma components (for example, ferrous citrate, hemoglobin, and thrombin) have been shown to involve in ICH-induced SBI (21). Iron-induced autophagy has been reported after ICH (22). Research in experimental autoimmune encephalomyelitis (EAE) models has demonstrated that α7nAChR can protect against neuroinflammation *via* autophagy in monocytes and microglia (23). Using an inflammatory bowel disease (IBD) model, it has been shown that α7nAChR deficiency exacerbates the severity of IBD in DSS-induced mice, while α7nAChR activation increases autophagy and suppresses proinflammatory cytokines in BMDM *via* LPS/DSS stimulation (24). It is unclear whether autophagy is involved in α7nAChR-mediated effects of intracerebral hemorrhage.

In this research, we hypothesized that activation of α7nAChR may reduce neurological and cardiac dysfunction *via* the induction of autophagy in the brain and heart following ICH. We aimed to investigate the pathogenesis of ICH and thereby provide novel insights for the development of therapeutic strategies for ICH.

## Methods and Materials

All experiments were performed according to the guidelines and approved by the National Institutes of Health guidelines for the Animal Care and Use Committee of Tianjin Medical University General Hospital. This study follows the guidelines to promote transparency and openness. All adequate measures were taken to minimize animal pain or discomfort.

### Experimental Groups

Adult male C57BL/6J mice (20–23g, 6–8 weeks) were obtained from Vital River Laboratory Animal Technology (Beijing, China). Mice were placed in a temperature-controlled environment for a 12-hour light-dark cycle with free access to food and water. We divided mice into five groups randomly **(1)** control **(2)**; ICH **(3)**; ICH + PNU-282987 **(4)**; ICH+PMN-282987+ (methyllycaconitine) MLA **(5)**; ICH+PNU-282987+3-MA. Mice were sacrificed at the following time points for investigations: 3 days after ICH to harvest brain tissue for brain water content; 7 days after ICH to obtain heart and brain tissue for real-time polymerase chain reaction (PCR), western blot, immunofluorescence and flow cytometry; and 30 days after ICH to harvest heart for real-time PCR and Picro-Sirius Red (PSR) staining. Echocardiographic assessment of cardiac function at 7 and 30 days after intracerebral hemorrhage.

### Drug Administration

The following drugs were used in the study: PNU-282987 (an α7nAChR agonist, 1 mg/kg/day, Abcam, ab120558) (25–27), Methyllycaconitine citrate (MLA, an α7nAChR antagonist, 1 mg/kg/day, Tocris Bioscience, 1029) (28–30) and 3-MA (an autophagy inhibitor, 10 mg/kg/day, MedChemExpress, HY-19312) (23). All drugs were injected intraperitoneally. Based on salt weight and concentration, compounds were dissolved in 0.9% saline, the injection volume is 1 ml/kg body weight. The first dose was administered 1 hour after ICH induction, followed by daily doses at 24-hour intervals. Control animals were injected with saline.

### ICH Model

We used a collagenase injection-induced intracerebral hemorrhage model. The procedure is easy to use and simulates intracerebral hemorrhage in humans (31). ICH in mice was induced by collagenase as described above. Mice were anesthetized using 5% chloral hydrate. After placing the mouse on the stereotaxic frame, drill a 1 mm hole on the right side of the skull (2.3 mm from lateral to the midline and 0.5 mm from the front of the bregma). In the collagenase induced ICH model, 0.0375 U of bacterial collagenase was dissolved in 0.5 µl saline solution and injected at a rate into the stratum (2.3 mm left, 0.5 mm anterior, 3.5 mm deep relative to bregma), the injection speed was 0.5 μl/min. After the injection was completed, leave the needle in place for 15 minutes to prevent backflow, then carefully removed at a speed of 1 mm/min.

### Neurological Function and Cognitive Function Tests

All functional tests were performed by an investigator who was blinded to the experimental group. The modified neurological severity score (mNSS) and foot-fault test were performed before and 1, 3, 7, 14, 21 days after the ICH to comprehensively estimate sensory, motor, reflex, and balance functions. The foot-fault test was used to evaluate the contralateral motor function deficit. Mice were photographed walking on the irregular grid for a period in a quiet environment. Contralateral limb foot faults percentage was counted. The higher score in this test, the more severe the neurological deficit.

### Brain Water Content

After 3 days of ICH, the mice were deeply anesthetized with 10% chloral hydrate. Their brains tissue was obtained and divided into three parts: the contralateral hemisphere, ipsilateral hemisphere, and cerebellum. Then, the tissue was weighed to wet weight and dried at 100°C for 24 hours to obtain dry weight. Calculate the water content in the brain using the formula: (wet weight - dry weight)/wet weight x 100%.

### Echocardiography Measurements

A researcher blinded to the experimental group performed echocardiography for non-invasive evaluation of cardiac function in mice using a Vevo2100 High-Resolution Ultrasound System in real-time (Visual Sonics Vevo 2100, Canada) with an MS-250 ultrasound scanning transducer (model C5). Anesthetized the mouse with 5% isoflurane at 0.5 L/min mixed with 100% O2 and place it on a heating pad. Use 1.0-1.5% isoflurane and 0.5 l/min 100% O2 to maintain sedation during surgery. Stabilized parasternal long-axis view images using m-mode imaging. Measure or calculate the following parameters: left ventricular fractional shortening (LVFS), left ventricular ejection fraction (LVEF), interventricular septum thickness in systole (IVS; s), interventricular septum thickness in diastole (IVS; d), left ventricle interior diameter in systole (LVID; s), left ventricle interior diameter in diastole (LVID; d), left ventricular volume in systole (LV volume; s) left ventricular volume in diastole (LV volume; d). Data were calculated using software on the ultrasound system in a blinded fashion.

### Pathological and Immunofluorescence Assessments

Heart and brain tissues were fixed in 5% paraformaldehyde for 24 hours and then embedded in paraffin or OCT. Cut coronal sections of brain and heart tissue to 7 µm. PSR staining was used to analyze the cross-sectional area of ​​cardiomyocytes and the composition of interstitial collagen. Tissue sections were stored at −80°C and rewarmed for 20 minutes then washed with phosphate-buffered saline (PBS) for 3 × 5 min. Brain and heart slides were incubated with 0.3% triton and 3% bovine serum albumin (BSA) dissolved in PBS for 90 minutes at room temperature (37°C). The following primary antibodies were used: rabbit anti-macrophage chemokine protein-1 (MCP-1, 1:200, Abcam), rabbit anti-NADPH oxidase-2 (NOX-2, Abcam, 1:250), rabbit anti- transforming growth factor-β (TGF-β, Santa Cruz Biotechnology, 1:250), rabbit anti-light chain 3(LC3, Cell Signaling Technology, 1:200), Antibodies were diluted with 0.3% triton and 3% BSA. Brain sections were conjugated with anti-LC3 overnight (12–14 h) at 4°C. Heart sections were treated with anti-TGF-β, anti-MCP-1, anti-NOX-2 and anti-LC3 overnight at 4°C. Then washed with cold PBS 3 x 10 min. Incubate slides with fluorophore-conjugated secondary antibodies for 1 hour respectively at room temperature. Sections were incubated with diamidino phenylindole (DAPI, nuclear staining, Abcam). Cover the slide with a coverslip and observe under a fluorescence microscope. Immunofluorescence quantification was performed on 4 sections, and heart and brain sections were measured by immunostaining in 5 random fields, selected using a fluorescence microscope (Olympus, Japan) under a 20x objective. Data analysis was performed using Image-Pro Plus 6.0 software.

### Quantitative Real-Time PCR

After ICH 7 days, RNA was isolated from the heart and brain using TRIzol reagent (Invitrogen) and quantitated by UV spectrophotometry at 260/280 nm. Complementary DNA (cDNA) was transcribed using the Transcription First Strand cDNA Synthesis SuperMix Kit (Transgen). PCR was operated on an Opticon 2 Real-Time PCR Detection System (Bio-Rad, Hercules, USA) with SYBR Green PCR Master Mix (Roche Diagnostics, Switzerland) and the primers. Samples were analyzed in normalized and duplicated to glyceraldehyde-3-phosphate dehydrogenase (GAPDH). The expression level of mRNA was calculated as a fold change compared to the control group. The primer sequences are as follows.

Arg-1(FWD:GAACACGGCAGTGGCTTTAAC;REV:TGCTTAGCTCTGTCTGCTTTGC);

YM-1(FWD:CGAGGTAATGAGTGGGTTGG;REV:CACGGCACCTCCTAAATTGT)

CD206(FWD:CAAGGAAGGTTGGCATTTGT;REV:CCTTTCAGTCCTTTGCAAGC);

CD86(FWD:TTGTGTGTGTTCTGGAAACGGAG;REV:AACTTAGAGGCTGTGTTGCTGGG);

GAPDH(FWD:GCCAAGGCTGTGGGCAAGGT;REV:TCTCCAGGCGGCACGTCAGA);

IL-1β(FWD:TCCAGGATGAGGACATGAGCAC;REV:GAACGTCACACACCAGCAGGTTA);

MCP-1:(FWD:CTGCTACTCATTCACCAGCAAG;REV:CTCTCTCTTGAGCTTGGTGACA);

TNF-α:(FWD:TACTCCCAGGTTCTCTTCAAGG;REV:GGAGGTTGACTTTCTCCTGGTA);

MMP-9:(FWD:AAACTCTTCTAGAGACTGGGAAGGAG;REV:AGCTGATTGACTAAAGTAGCTGGA);

TLR2:(FWD:TTATCTTGCGCAGTTTGCAG;REV:CTCCCACTTCAGGCTCTTTG);

VCAM:(FWD:CTGGGAAGCTGGAACGAAGT;REV:GCCAAACACTTGACCGTGAC);

ZO-1:(FWD:TGAACGTCCCTGACCTTTCG;REV:CTGTGGAGACTGCGTGGAAT);

Claudin-5:(FWD:GTTAAGGCACGGGTAGCACT;REV:TACTTCTGTGACACCGGCAC);

Occludin:(FWD:TGGCAAAGTGAATGGCAAGC;REV:TCATAGTGGTCAGGGTCCGT);

α7nAChR:(FWD:GTCCTGGTCCTATGGAGGGT;REV:AGGGCTGAAATGAGCACACA);

HMGB1:(FWD:AGTGGCTTTTGTCCCTCATCC;REV:GGACATTTTGCCTCTCGGCT).

### Western Blot

Cell lysates with equal amounts were extracted from the heart and brain samples for Western blot analysis. Protein concentration was measured using the bicinchoninic acid method (Thermo Scientific). Samples were loaded at 12% Tris glycine gels, subjected to sodium dodecyl sulfate–polyacrylamide gel electrophoresis, and transferred onto polyvinylidene difluoride membranes (Millipore, USA). The membrane was blocked with 5% non-fat dry milk for 2 hours and incubated with primary antibody overnight at 4°C. Immunoblotting was conducted using rabbit anti-LC3 antibody (1:1000, Cell Signaling Technology, 2775S), rabbit anti-Beclin antibody (1:1000, Cell Signaling Technology, 3495S), rabbit anti-p62/SQSTM1 antibody (1:1000, Cell Signaling Technology, 5114S), rat anti-a7nAChR (1:200 Santa Cruz Biotechnology, sc-58607), mouse anti-GAPDH antibody (1:1000, Nakasugi Jinqiao). The membranes were then incubated with species-appropriate secondary antibodies for 1 hour at room temperature. The protein bands were visualized with a Bio-Rad Gel Imager.

### Flow Cytometry

The mice were sacrificed 7 days after the intracerebral hemorrhage and brain tissue was collected to prepare a single cell suspension. In brief, for all animals, the brains were removed, minced with scissors, incubated with collagenase D4 (Sigma-Aldrich, 1 mg/ml) and Dispase (Roche, 1 mg/ml) and digested at 37°C for 60 minutes. The homogenate was passed through a 70μm filter and the filtrate was centrifuged for 3 min at 2000 rpm. The centrifuged cells were resuspended in 30% Percoll and centrifuged for 10 min at 700 × g. The particles were washed with cold PBS for 10 minutes at 2000 rpm. Incubate the single cell suspension with the following fluorescently labeled antibodies: CD11b-FITC, CD45-Percp, F4/80-APC, CD206-PE/cy7, CD86-PE and Ly6G-APC/cy7, (BioLegend, San Diego, USA). The data of flow cytometry were acquired on a FACSAriaTM Flow cytometer (BD Biosciences, San Jose, USA) and analyzed with FlowJo software.

### Statistical Analysis

Statistics were analyzed by Graphpad Prism. Statistical analysis was conducted by unpaired 2-tailed Student t-test for comparison of two groups, one-way analysis of variance (Tukey) was used for analysis among the five groups and repeated measure analysis of variance was used to study the difference in foot-fault functional and mNSS tests. All values are expressed as mean ± standard error, p values < 0.05 are considered statistically significant.

## Results

### Activating α7nAChR Decreased Brain Water Content 3 Days After ICH

**Figure 1:**
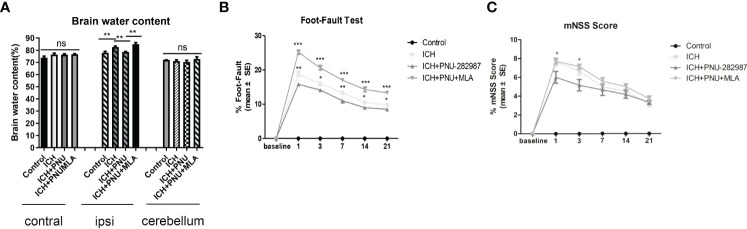
Activating α7nAChR decreased brain water content 3 days after ICH and improves neurological function in ICH mice. **(A)** PNU-282987 significantly reduces water content in the brain in the ipsilateral hemisphere. **(B)** Activating α7nAChR decreases foot-fault test scores in ICH mice. **(C)** Activating α7nAChR decreases mNSS scores in ICH mice. n= 5/group, the results are expressed as mean ± SE, *p < 0.05, **P < 0.01, ***P < 0.0001, ns means p > 0.05.

To determine whether treatment with the α7nAChR agonist PNU-282987 reduces brain water content during the acute stage of ICH, brain water content was evaluated at 3 days after surgery. **Figure 1A** shows that PNU-282987 remarkably decreased water content in the ipsilateral cerebral hemisphere compared to ICH + saline group. However, MLA reversed this effect entirely. There is no significant difference between the contralateral hemisphere and the cerebellum.

### Activating α7nAChR Improved Neurological Function in ICH Mice

A series of neurological and cognitive tests were performed to test the effects of PNU-282987 on neurological deficits. The foot-fault test and the mNSS were performed after ICH. The data showed that treatment with PNU-282987 reduce neurological deficits in the ICH + PNU-282987 treatment group compared to the ICH + saline group, while this treatment effect was reversed by the administration of MLA (**Figures 1B, C**).

### Activating α7nAChR Prevented ICH-Induced Cardiac Dysfunction

**Figure 2:**
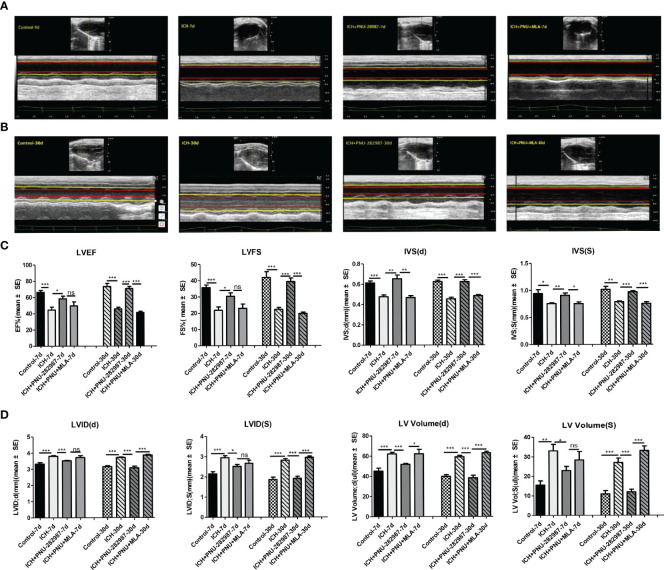
Activating α7nAChR prevented ICH-induced cardiac dysfunction. **(A)** Representative echocardiography results at 7 days from control, ICH, ICH+PNU-282987, ICH+PNU-282987+MLA. **(B)** Representative echocardiography results at 30 days. **(C)** LVEF, LVFS dimension at 7 days and 30 days after ICH, IVS dimension at the end of diastole and systole. **(D)** LV volume, LVID dimension at the end of diastole and systole. n = 6 per group for echocardiography. Data are presented as mean ± SE; *p < 0.05; **P < 0.01; ***P < 0.0001; ns means p > 0.05.

Echocardiography was employed to test whether the cholinergic anti-inflammatory pathway ameliorated ICH-induced cardiac dysfunction. Data in **Figures 2A, B** presents typical echocardiographic data at 7 and 30 days after ICH. These data revealed that ICH induced acute (7 days) and chronic (30 days) cardiac dysfunction in mice illustrated by reduced LVEF, LVFS and IVS (d, s) compared to control group. PNU-282987 treatment remarkably improved systolic function by increasing LVEF, LVFS and IVS (d, s) at 7 and 30 days after ICH. However, MLA could reverse this effect at 30 days after ICH (**Figure 2C**). It also exhibits significant increases in LVID (d, s) and LV volume (d,s) in ICH mice at 7 and 30 days after ICH compared with controls. The treatment of PNU-282987 decreases LVID and LV volume at 7 days and 30 days, whereas MLA reversed this effect (**Figure 2D**). Our data suggest that ICH may lead to significant disturbances in cardiac function in both acute and chronic phases, PNU-282987 significantly attenuates myocardial dysfunction induced by ICH.

### Activating α7nAChR Significantly Decreased Cardiac Fibrosis and Cardiac Hypertrophy in the Chronic Phase of ICH

**Figure 3:**
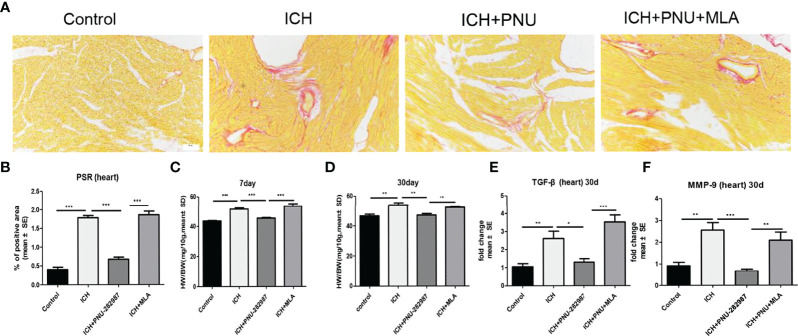
Activating α7nAChR significantly decreased cardiac fibrosis and cardiac hypertrophy in the chronic phase of intracerebral hemorrhage. **(A)** Representative results for PSR immunostaining in heart tissue at 30 days. **(B)** Quantification of interstitial fibrosis (Picro Sirius Red). n = 6 per group. **(C)** The result of heart weight at 7 days. **(D)** The result of heart weight at 30 days. **(E)** The PCR results showed that treatment with PNU-282987 significantly reduced the gene expression of the ICH-caused profibrotic factor TGF-β compared with ICH mice at 30 days. **(F)** PNU-282987 significantly reduced the gene expression of the ICH-caused profibrotic factor MMP-9 compared with ICH mice at 30 days. n = 6 per group. Data are presented as mean ± SE; *p < 0.05; **P < 0.01; ***P < 0.0001.

In our previous studies, we discovered that ICH led to cardiac fibrosis and myocyte hypertrophy in the chronic phase of ICH. To observe the effect of α7nAChR on the heart, we detected myocardial fibrosis and cardiomyocytes using PSR 30 days after ICH. Cardiac hypertrophy was evaluated as heart weight to body weight ratio (mg/10 g). **Figures 3A–D** shows that ICH induced myocardial hypertrophy and fibrosis compared to control. PNU-282987 decreases cardiac hypertrophy and fibrosis compared to ICH mice. However, MLA reverses this effect. TGF-β has been proven to play a vital role in mediating cardiac fibrosis (32). Matrix metalloproteinase-9 (MMP-9) is used as a marker for changes in the heart associated with inflammation and fibrosis (33, 34). The PCR data of TGF-β and MMP-9 indicates that ICH significantly increases the level of TGF-β and MMP-9 expression in the heart compared to control mice in the chronic phase of ICH. PNU-282987 significantly decreased the level of TGF-β and MMP-9 expression compared to ICH mice, contrary to MLA’s effect (**Figures 3E, F**). Therefore, it is concluded that activating α7nAChR significantly decreased cardiac fibrosis and cardiac hypertrophy during the chronic phase of ICH.

### Activating α7nAChR Induced Autophagy in the Brain and Heart of ICH Mice

**Figure 4:**
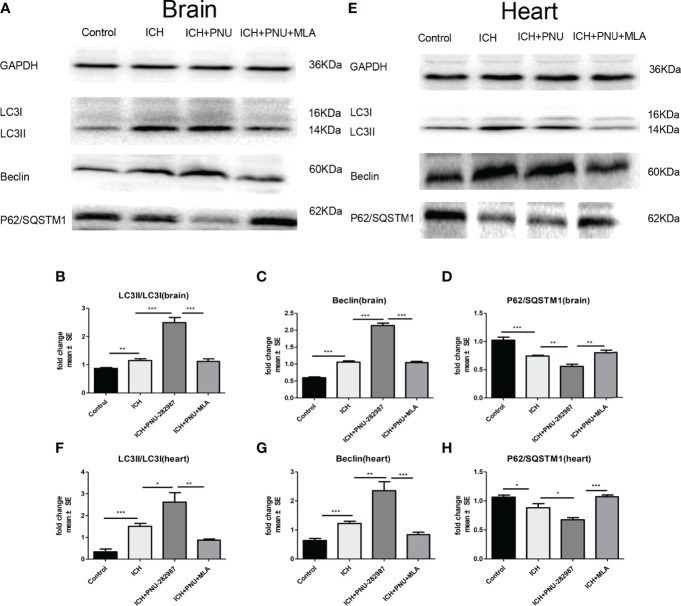
Activating α7nAChR induced autophagy in the brain and heart of ICH mice. **(A)** Representative protein expression levels of LC3, Beclin, p62/SQSTM1 in brain evaluated by western blot. **(B)** Quantification of western blot data of LC3 in brain. **(C)** Quantification of western blot data of Beclin in brain. **(D)** Quantification of western blot data of p62/SQSTM1 in brain. **(E)** Representative protein expression levels of LC3, Beclin, p62/SQSTM1 in heart, as measured by western blot. **(F)** Quantification of western blot data of LC3 in heart. **(G)** Quantification of western blot data of Beclin in heart. **(H)** Quantification of western blot data of p62/SQSTM1 in heart. n= 6 per group. Data are presented as mean ± SE; *p < 0.05; **P < 0.01; ***P < 0.0001.

**Figure 5:**
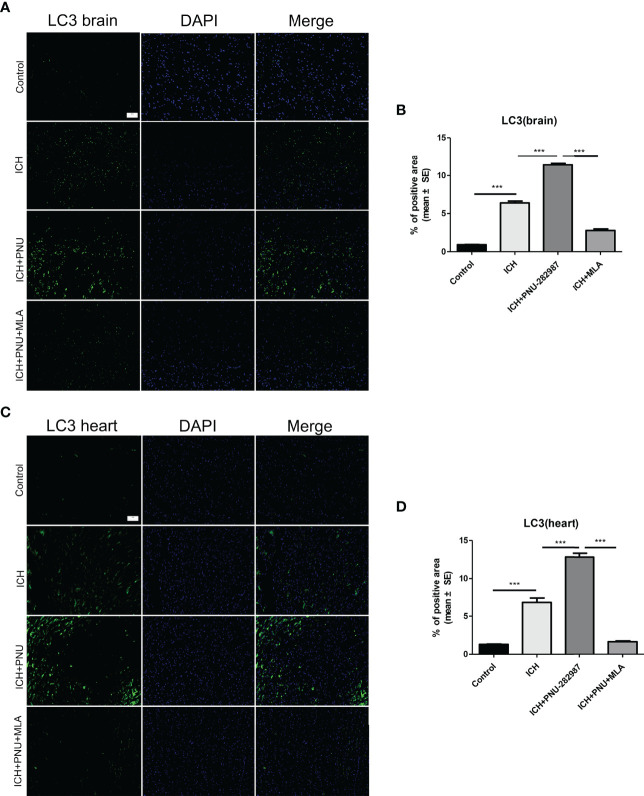
Activating α7nAChR induced autophagy in the brain and heart of ICH mice. **(A)** Immunofluorescence staining of LC3 from brain tissue of control, ICH, ICH+PNU-282987 and ICH+PNU-282987+MLA (scale bar, 100 μm). **(B)** The quantitation of LC3 in brain. **(C)** Immunofluorescence staining of LC3 from heart tissue of control, ICH, ICH+PNU-282987 and ICH+PNU-282987+MLA (scale bar, 100 μm). **(D)** The quantitation of LC3 in heart. n= 6 per group. Data are presented as mean ± SE; ***P < 0.0001.

Previous studies have shown that autophagy plays a controversial role in ICH by regulating the production of inflammatory cytokines (35). Therefore, we questioned whether the protective effect of α7nAChR activation on ICH was related to enhanced autophagy. Firstly, we used immunoblotting to test the effect of activated α7nAChR on the autophagy-related protein in the brain and heart of ICH mice. We found that activated α7nAChR by PNU-282987 significantly increased the expression level of LC3-II/I ratio and the Beclin, while reduced the expression of p62/SQSTM1 in the brain and heart after ICH. (**Figures 4A–H**). Secondly, we conducted further immunofluorescence staining of LC3 in the heart and brain. We found that ICH mice exhibited higher expression level of LC3 in the brain and heart compared to controls. PNU-282987 treatment could further increase the expression of LC3, while MLA blocked these effects (**Figures 5A–D**). Collectively, these data suggest that α7nAChR activation promotes autophagy in the brain and heart after ICH.

### The Expression of a7nAChR Was Decreased in Brain and Heart After ICH

**Figure 6:**
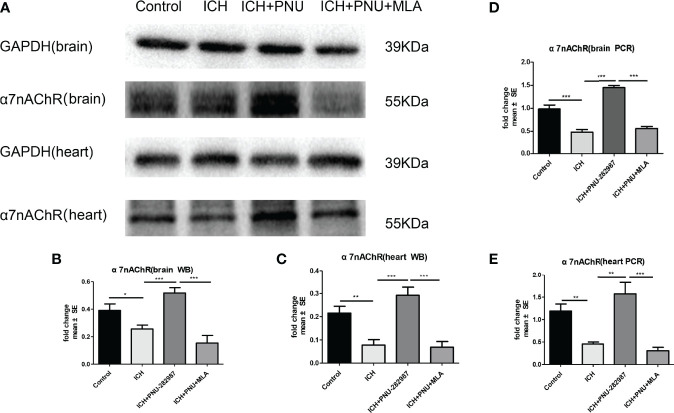
The expression of α7nAChR was reduced in brain and heart After ICH. **(A)** Representative protein expression levels of α7nAChR in brain and heart, the expression of protein α7nAChR remarkably reduced at 7 days after ICH, while PNU-282987 promoted their expression. **(B)** Quantification of western blot data in brain. **(C)** Quantification of western blot data in heart. **(D)** ICH for 7 days significantly decreased mRNA expression of α7nAChR in brain, PNU-282987 promoted their expression. **(E)** ICH for 7 days significantly decreased mRNA expression of α7nAChR in heart, PNU-282987 promoted their expression. n= 6 per group. Data are presented as mean ± SE; *p < 0.05; **P < 0.01; ***P < 0.0001.

PCR and Western blotting were used to measure the expression of α7nAChR for 7 days after ICH. The data showed that protein expression of α7nAChR after ICH was decreased compared to control mice (**Figures 6A–C**), as well as the mRNA expression of a7nAChR (**Figures 6D, E**). These results demonstrate that PNU-282987 increases the expression of α7nAChR in the brain and heart of mice after ICH.

### Activating α7nAChR Mediated Neuroprotection in ICH by Regulating Microglia/Macrophage Polarization Toward to an Anti-Inflammatory Phenotype

**Figure 7:**
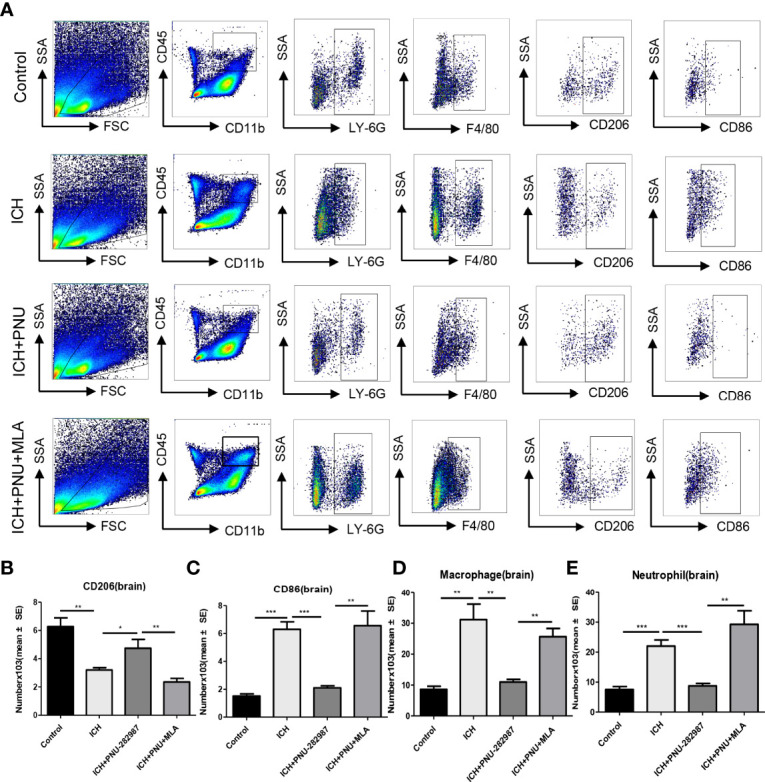
Activating α7nAChR mediated neuroprotection in ICH by regulating microglia/macrophage polarization toward an anti-inflammatory phenotype. **(A)** Typical flow cytometric graph showing the gating strategy for CD45+CD11b+F4/80+ macrophages, CD45+CD11b+Ly6G+neutrophils, CD206+ anti-inflammatory macrophages and CD86+ pro-inflammatory macrophages populations in brain. **(B)** Quantitative data of anti-inflammatory macrophages expression in brain at 7 days after ICH. **(C)** Quantitative data of pro-inflammatory macrophages expression in brain at 7 days after ICH. **(D)** Quantitative data of macrophages expression in brain at 7 days after ICH. n = 6 per group. **(E)** Quantitative data of neutrophils expression in brain at 7 days after ICH. Data are presented as mean ± SE; *p < 0.05; **P < 0.01; ***P < 0.0001.

**Figure 8:**
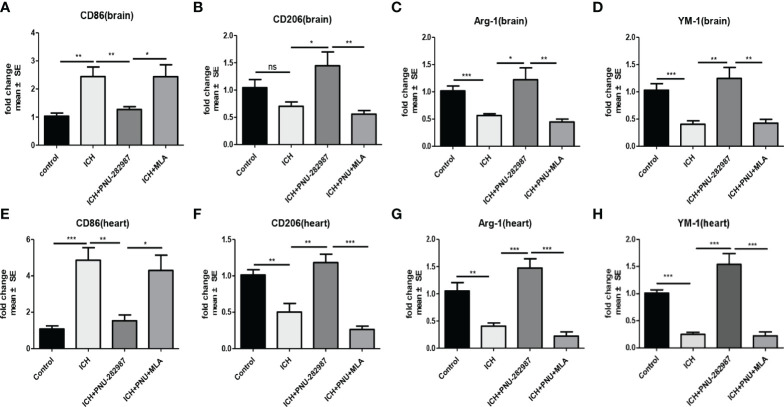
The results of PCR demonstrated that PNU-282987 drastically decreased the phenotype of pro-inflammatory macrophages [CD86 **(A)**] and increased anti-inflammatory macrophages [CD206 **(B)**, Arg-1 **(C)**, YM-1 **(D)**] in brain. PCR results indicated that PNU-282987 significantly reduced the pro-inflammatory macrophages [CD86 **(E)**] and increased the phenotype of anti-inflammatory macrophages [CD206 **(F)**, Arg-1 **(G)**, YM-1 **(H)**] in heart. n= 6 per group for echocardiography. Data are presented as mean ± SE; *p < 0.05; **P < 0.01; ***P < 0.0001; ns means p > 0.05.

Macrophage/microglia can polarize into two phenotypes: anti-inflammatory and pro-inflammatory, producing anti- or pro-inflammatory chemokines and cytokines (36, 37). Analyse of whether activating α7nAChR alters microglia/macrophage polarization, we performed flow cytometry to measure the expression of CD206 and CD86 in the brain for 7 days. We quantified the expression of pro-inflammatory markers (CD86) and anti-inflammatory markers (CD206, YM-1 and Arg-1) in brain and heart for 7 days using real-time PCR to confirm flow cytometry results. The data showed that the expression of anti-inflammatory macrophages was decreased in ICH mice compared to the control mice. PNU-282987 treatment increased anti-inflammatory macrophages expression. However, this effect was completely reversed by co-administration of MLA. The expression of pro-inflammatory macrophages was increased in ICH mice compared to the control mice. PNU-282987 treatment decreased pro-inflammatory macrophages expression. Again, the effect was completely reversed by MLA (**Figures 7A–C**, **8A–H**).

### α7nAChR Expression in Endothelial Cells Promoted Angiogenesis and Alleviated the Functional Dysfunction of the Blood–Brain Barrier After ICH

**Figure 9:**
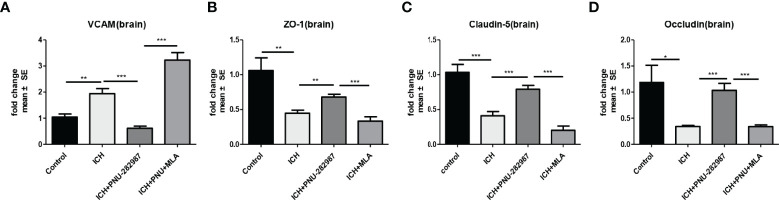
α7nAChR expression in endothelial cells promoted angiogenesis and alleviated the functional dysfunction of the blood–brain barrier after ICH. **(A)** PCR results indicated that PNU-282987 remarkably decreased the expression of VCAM. **(B)** The results of PCR demonstrated that PNU-282987 drastically increased the expression of ZO-1. **(C)** The results of PCR demonstrated that PNU-282987 drastically increased the expression of Claudin-5. **(D)** The results of PCR demonstrated that PNU-282987 drastically increased the expression of Occludin. n= 6 per group for echocardiography. Data are presented as mean ± SE; *p < 0.05; **P < 0.01; ***P < 0.0001.

α7nAChR is broadly expressed in the central and peripheral nervous systems and also in non-neuronal tissues such as endothelial cells. Activation of endothelial cells is characterized by increased expression of adhesion molecules on the cell surface (38). VCAM plays an important role in this process. In our experiment, we observed that PNU-282987 significantly increased VCAM mRNA expression (**Figure 9A**). Furthermore, the mRNA expression of ZO-1, Claudin-5, Occludin was increased by PNU-282987. However, the effects were completely reversed by MLA (**Figures 9B–D**). These data showed that activated α7nAChR significantly induced endothelial cell activation, promoted angiogenesis and restored the function of complex blood brain barrier.

### Activating α7nAChR Decreased ICH-Caused Oxidative Stress and Inflammatory Responses in the Brain and Heart

**Figure 10:**
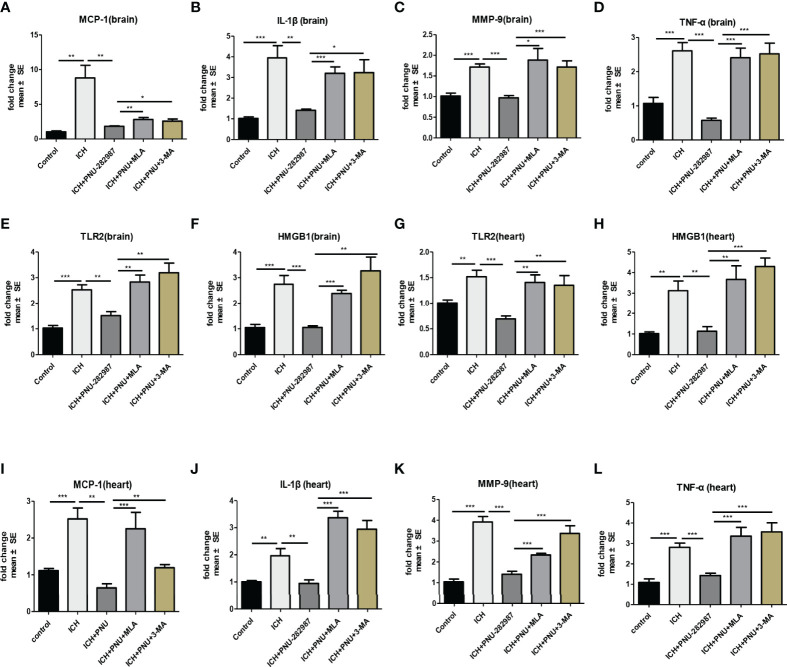
Activating α7nAChR decreased ICH-caused oxidative stress and inflammatory responses in the brain and heart. **(A–F)** PCR results indicated that PNU-282987 remarkably decreased ICH-induced inflammatory factor expression as MCP-1, IL-β, MMP-9, TNF-α, TLR2 and HMGB1 compared to control mice in brain at 7 days after ICH. **(G–L)** PCR results indicated that PNU-282987 significantly reduced gene expression of ICH-induced inflammatory factor as TLR2, HMGB1, MCP-1, IL-β, MMP-9 and TNF-α compared to control mice in heart at 7 days after ICH. n = 6 per group for echocardiography. Data are presented as mean ± SE; *p < 0.05; **P < 0.01; ***P < 0.0001.

**Figure 11:**
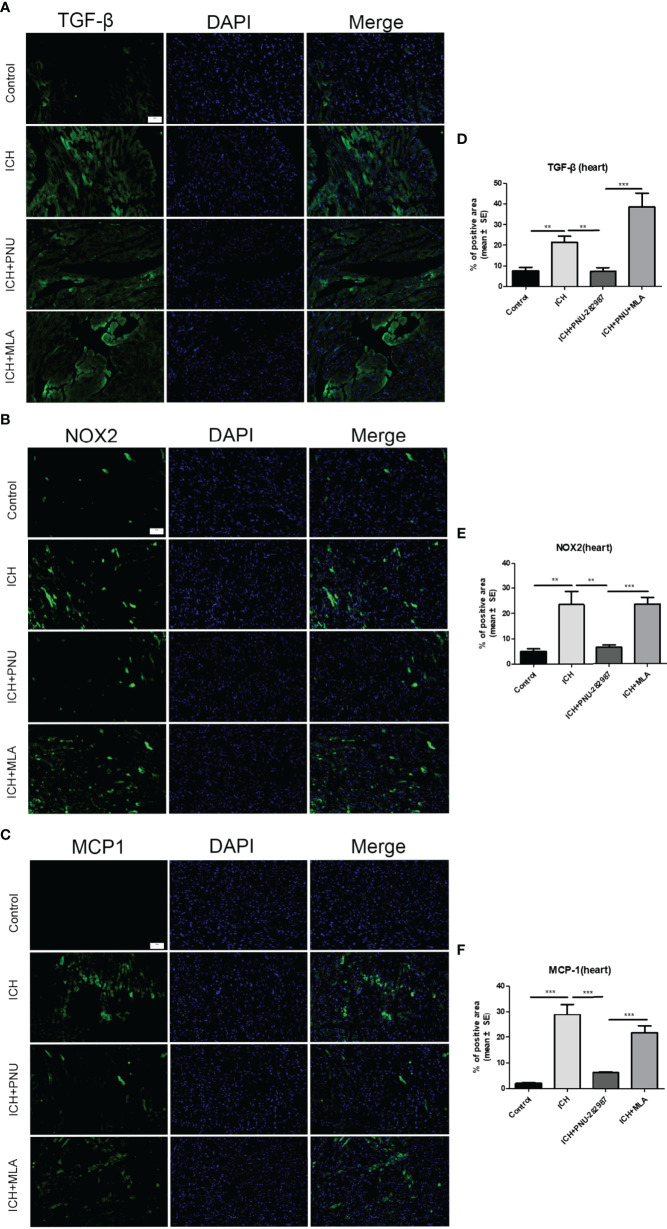
Activating α7nAChR decreased ICH-caused oxidative stress and inflammatory responses in the heart. Immunofluorescence images of inflammation factor of TGF-β **(A)**, NOX-2 **(B)**, MCP-1 **(C)** were compared in four groups in heart, (scale bar, 100 μm). Quantitative data of TGF-β **(D)**, NOX-2 **(E)**, MCP-1 **(F)** in heart. n = 6 per group. Data are presented as mean ± SE; **P < 0.01; ***P < 0.0001.

In our previous studies, it was confirmed that ICH induced cardiac inflammation (9). The mRNA expression of inflammatory factor (IL-1β, TNF-α, MCP-1, HMGB1, MMP-9 and TLR2) were measured by PCR in brain (**Figures 10A–F**) and heart (**Figures 10G–L**) tissue harvested at 7 days after ICH. To corroborate the PCR findings, we used immunofluorescence staining to measure NOX-2, MCP-1 and TGF-β in heart tissue (**Figures 11A–F**). The results showed that the inflammatory factor increased in ICH mice compared to control. PNU-282987 treatment decreased inflammatory factor expression. However, this effect was completely reversed by MLA and 3-MA. We also performed flow cytometry to measure the expression of neutrophils (CD45+ CD11b+ Ly6G+) and macrophages (CD45+ CD11b+ F4/80+). As demonstrated in **Figures 7A, D–E**, macrophages and neutrophils expression was significantly increased in ICH mice compared to control. Furthermore, macrophages and neutrophils were significantly reduced in the brains of PNU-282987-treated ICH mice compared with saline-treated ICH mice. However, MLA blocked these effects. The results suggest that activation α7nAChR reduced inflammatory cells infiltration into the brain after ICH.

## Discussion

We have two main conclusions from this study. First, we demonstrate that α7nAChR activation protected against ICH injury through improving neurological and cardiac dysfunction. Second, our data provided direct evidence that autophagy is causally linked to α7nAChR activation and helped to ameliorate injury. It is widely believed that the α7nAChR is involved in a systemic anti-inflammatory mechanism called the cholinergic anti-inflammatory pathway (CAIP) (39). The CAIP has recently been described as a neuroimmunomodulatory pathway whose activation may be effective in reducing the release of pro-inflammatory cytokines and suppressing inflammatory response (40, 41). The cholinergic anti-inflammatory pathway acted primarily role through stimulation of the vagus nerve in an α7nAChR-dependent manner. In macrophages, α7nAChR activation inhibited pro-inflammatory transformation and promoted anti-inflammatory polarization, thereby regulating the inflammatory response (42).

It has been demonstrated previously that activating α7nAChR played a protective effect in a variety of cardiovascular and cerebrovascular diseases, including TBI, ischemic stroke, ICH, SAH, hypertension and myocardial ischemia (43–45). In our study, The experiment result showed that PNU-292987 administered 1 h after surgery could reduce the brain edema in the ipsilateral hemisphere 3 days after ICH, This finding coincides with Krafft et al. study, which indicated that intraperitoneal injection of PNU-282987 at a dose of 12mg/kg after ICH induction 1h could prevent to the increase in basal ganglia water content in male CD-1 mice at 3 days (15). While Hijioka et al. showed that PNU-282987 had no significant effect on the degree of hematoma and the formation of brain edema. The discrepancy may be due to different animal models, drug doses and times of drug administration (16). Whether PNU-282987 contributes to relieve the severity of brain edema warrants further studies. A study using a permanent middle cerebral artery occlusion model demonstrated that the neuroprotective effects of activating α7nAChR on the anti-oxidative stress, reduction of pro-inflammatory and NF-kB activity in macrophages/microglia (46). Furthermore, the α7nAChR agonist PHA-543613 attenuated neuroinflammation in rodents after experimental intracerebral hemorrhage by activating the JAK2-STAT3 signaling pathway (47). Other studies have shown that electroacupuncture stimulation induced significant neuroprotection in neurons after transient ischemic brain injury by modulating α7nAChR to inhibit the inflammatory response associated with NLRP3 inflammation, reduce apoptosis, and modulate the balance between anti- and pro-inflammatory factors (48).

In addition to cardiovascular and cerebrovascular diseases, α7nAChR also played a significant role in other systemic diseases. For instance, it has been demonstrated that the hepatic vagus nerve played a key role in regulating the inflammatory response of Kupffer cells *via* the α7nAChR pathway and ultimately inhibited the progression of nonalcoholic steatohepatitis at an early stage (49). Peng Teng demonstrated that stimulation of α7nAChR by nicotine attenuated monosodium iodoacetate-induced cartilage degradation and osteoarthritis pain, this protective effect of nicotine is associated with the inhibition of MMP-9 overexpression *via* the PI3K/Akt/NF-kB signaling pathway (50).

The various underlying mechanism of the cholinergic anti-inflammatory pathway after stroke has been extensively studied by several groups (51). A study showed that activation of α7nAChR and subsequent cholinergic anti-inflammatory signaling inhibited NF-κB nuclear translocation into macrophages, hence inhibiting the secretion of high mobility group box 1 (HMGB1), an important pro-inflammatory factors and mediator of advanced sepsis (40). Peña et al. demonstrated a new perspective to study the mechanism of cholinergic regulation of inflammation that is attributed to the JAK2/STAT3 signaling cascade. In their study, phosphorylated and unphosphorylated STAT3 were responsible for the anti-inflammatory result (52). Recent researches have also further elucidated alternative mechanisms of cholinergic control of inflammation involving PI3K signaling. Nicotine contributed to an increase in IRAK-M in macrophages *via* a cascade (JAK2/PI3K/STAT3) or both (JAK2/STAT3 and PI3K/STAT3) cascades. The anti-inflammatory effect of α7nAChR agonists is due to increased activity of IRAK-M, which has an anti-inflammatory activity (53). Another possible mechanism by which cholinergic modulates pro-inflammatory responses are to increase heme oxygenase 1 (HO-1) activity (53).

In this study, we determined the mechanism of the cholinergic anti-inflammatory pathway in ICH and its role in the therapeutic. We established a collagenase-induced ICH model in mice and determined that the α7nAchR agonist PNU-282987 can promote the transformation of macrophages from pro-inflammatory subtype to anti-inflammatory subtype, resulting in inhibition of inflammatory factors to improve neurological and cardiac dysfunction.

Autophagy is a conserved intracellular mechanism that mediated lipid aggregation, degradation of misfolded proteins and damaged organelles. In response to cellular stressors such as starvation and oxidative stress, autophagy is activated to limit cell death. In recent years, the role of autophagy in different neuronal disease models has attracted much attention. As is well known, autophagy is a “double-edged sword”. It was unclear whether autophagy played a beneficial or detrimental role. Some studies demonstrated that impaired autophagic currents are associated with neuronal cell death following TBI (54). Activation of the autophagy pathway *via* an anti-apoptotic mechanism reduced early brain injury in a rat model of SAH (55). Whereas other researchers have shown that autophagy can cause to cell death in focal cerebral ischemia models (56). Furthermore, one study suggested that activation of autophagy pathways played a neuroprotective role in Alzheimer’s disease, which is related to α7nAChR signaling (57). The relationship between induction of neuronal autophagy and activation of α7nAChR has been proven (58–60). Consistent with these findings, our results indicated that PNU-282987, an α7nAChR agonist promoted autophagy, which increased the Beclin, LC3-II/LC3-I ratio and reduced p62/SQSTM1, a marker of autophagic currents activated in ICH model. In our study, activation of α7nAChR promoted autophagy, reduced the release of inflammatory factors, and improved brain function and heart function. Administration of 3-MA, which inhibited class III PI3K and comprehensively acted as an autophagy inhibitor, reversed the effect of α7nAChR agonist on autophagy after ICH, indicated that the anti-inflammatory effect of α7nAChR is related to the autophagy pathway. However, whether the anti-inflammatory effect caused by promoting autophagy is caused by reducing the death of neurons or cardiomyocytes remains to be determined by further experiments.Whether activation of α7nAChR reduced or promoted autophagy remains contentious, as its role is distinct in diverse disease models. A publication has revealed that α7nAChR promoted protective autophagy in the LPS-induced mouse acute lung injury model and in MH-S cells (61). Li Z. et al. have also demonstrated that nicotine induced autophagy through the α7nAChR-mediated JAK2/STAT3 signaling pathway, thereby promoting the activation of human pancreatic stellate cells (62).

In our research, we found that PNU-282987, a positive allosteric modulator selective for α7nAChR, exerted protective effects against ICH damage by modulating the crosstalk between autophagy and inflammation. Although the use of PNU-282987 is limited, this study provides new evidence to support future development of therapeutic strategies for inflammatory diseases such as ICH *via* the cholinergic anti-inflammatory pathway.

## Data Availability Statement

The original contributions presented in the study are included in the article/**Supplementary Material**. Further inquiries can be directed to the corresponding author.

## Ethics Statement

The animal study was reviewed and approved by the Animal Care and Use Committee of Tianjin Medical University General Hospital.

## Author Contributions

TY: experimental design and gave final approval of manuscript. YS: experimental design, wrote the manuscript, performed experiments, analyzed data and prepared figures. WZ: performed experiments, analyzed data, prepared figures. RZ: experimental design and wrote the manuscript. QY, RW, XL, JW, and RL: performed experiments. All authors contributed to the article and approved the submitted version.

## Funding

This work was supported by National Natural Science Foundation of China (grant 82171320).

## Conflict of Interest

The authors declare that the research was conducted in the absence of any commercial or financial relationships that could be construed as a potential conflict of interest.

## Publisher’s Note

All claims expressed in this article are solely those of the authors and do not necessarily represent those of their affiliated organizations, or those of the publisher, the editors and the reviewers. Any product that may be evaluated in this article, or claim that may be made by its manufacturer, is not guaranteed or endorsed by the publisher.
