# Supplementary figures and images for: Activation of Cholinergic Anti-Inflammatory Pathway Ameliorates Cerebral and Cardiac Dysfunction After Intracerebral Hemorrhage Through Autophagy
Source: Front Immunol. 2022 Jun 23;13:870174. doi: 10.3389/fimmu.2022.870174 (PMC9260497; doi:10.3389/fimmu.2022.870174)

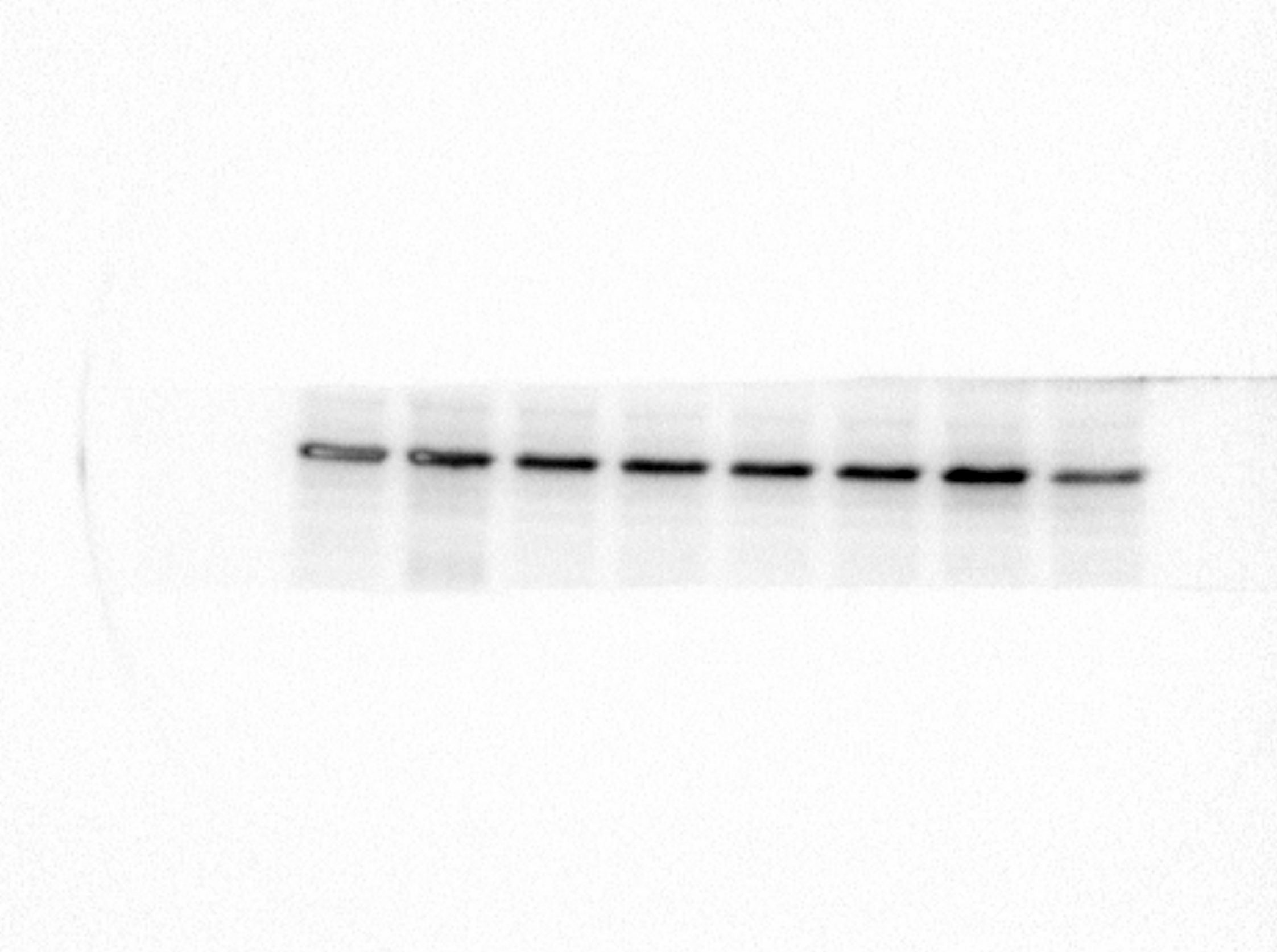

Supplement: Supplementary file 1 [file DataSheet_1.zip › supplementary materials (870174)/GAPDH brain-1.tif]

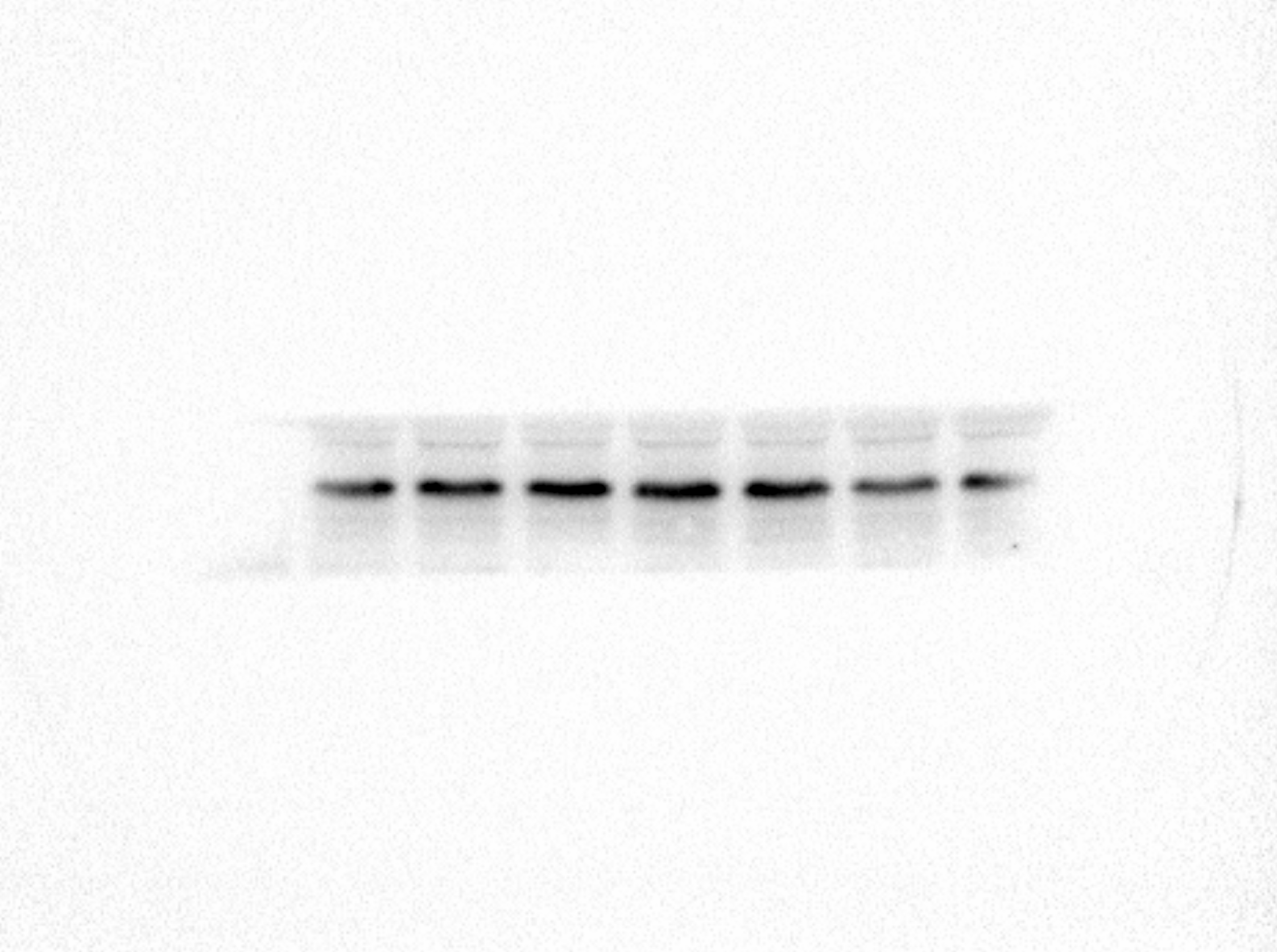

Supplement: Supplementary file 1 [file DataSheet_1.zip › supplementary materials (870174)/GAPDH heart-1.tif]

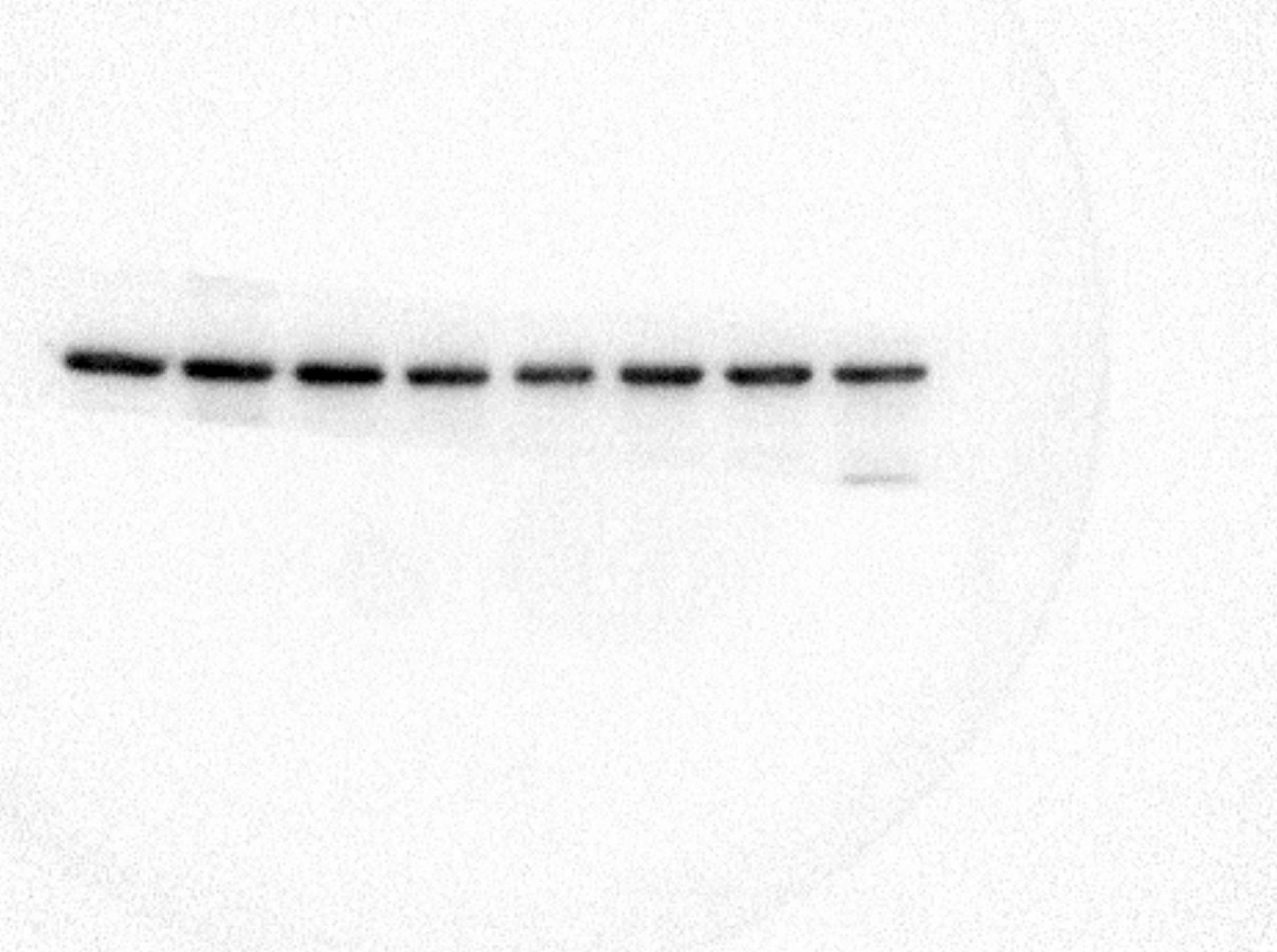

Supplement: Supplementary file 1 [file DataSheet_1.zip › supplementary materials (870174)/GAPDH-brain-2.tif]

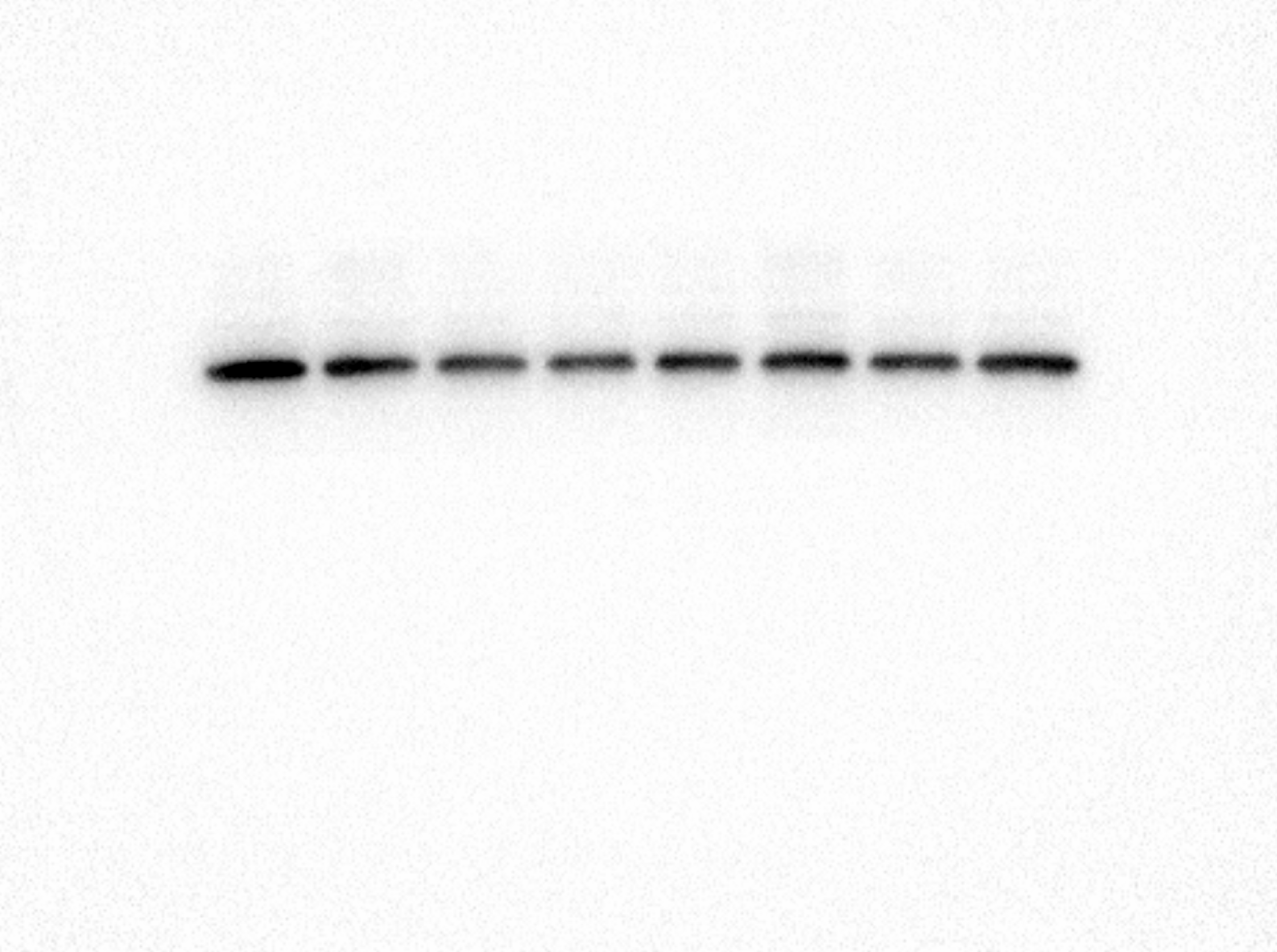

Supplement: Supplementary file 1 [file DataSheet_1.zip › supplementary materials (870174)/GAPDH-heart-2.tif]

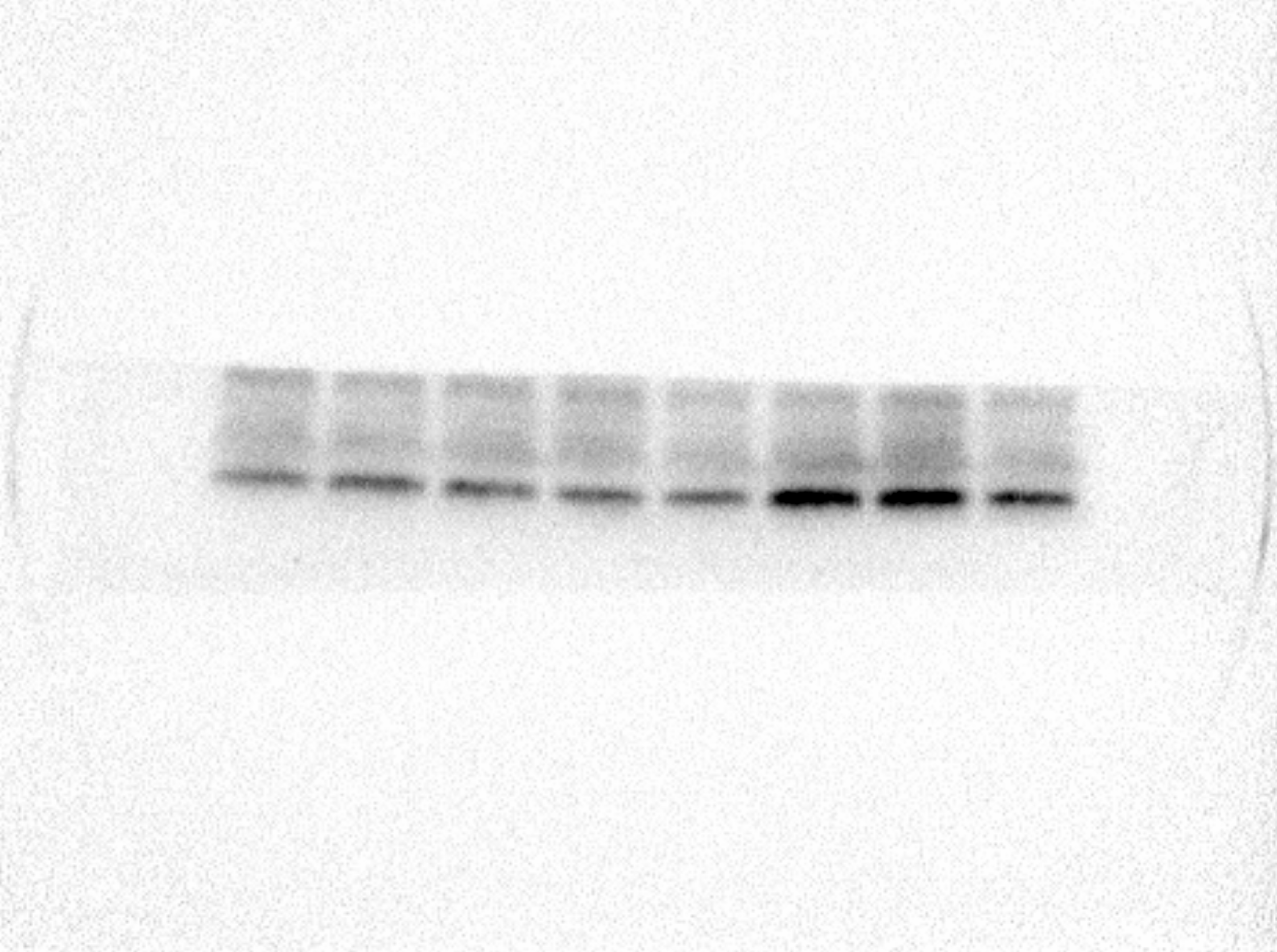

Supplement: Supplementary file 1 [file DataSheet_1.zip › supplementary materials (870174)/LC3 brain.tif]

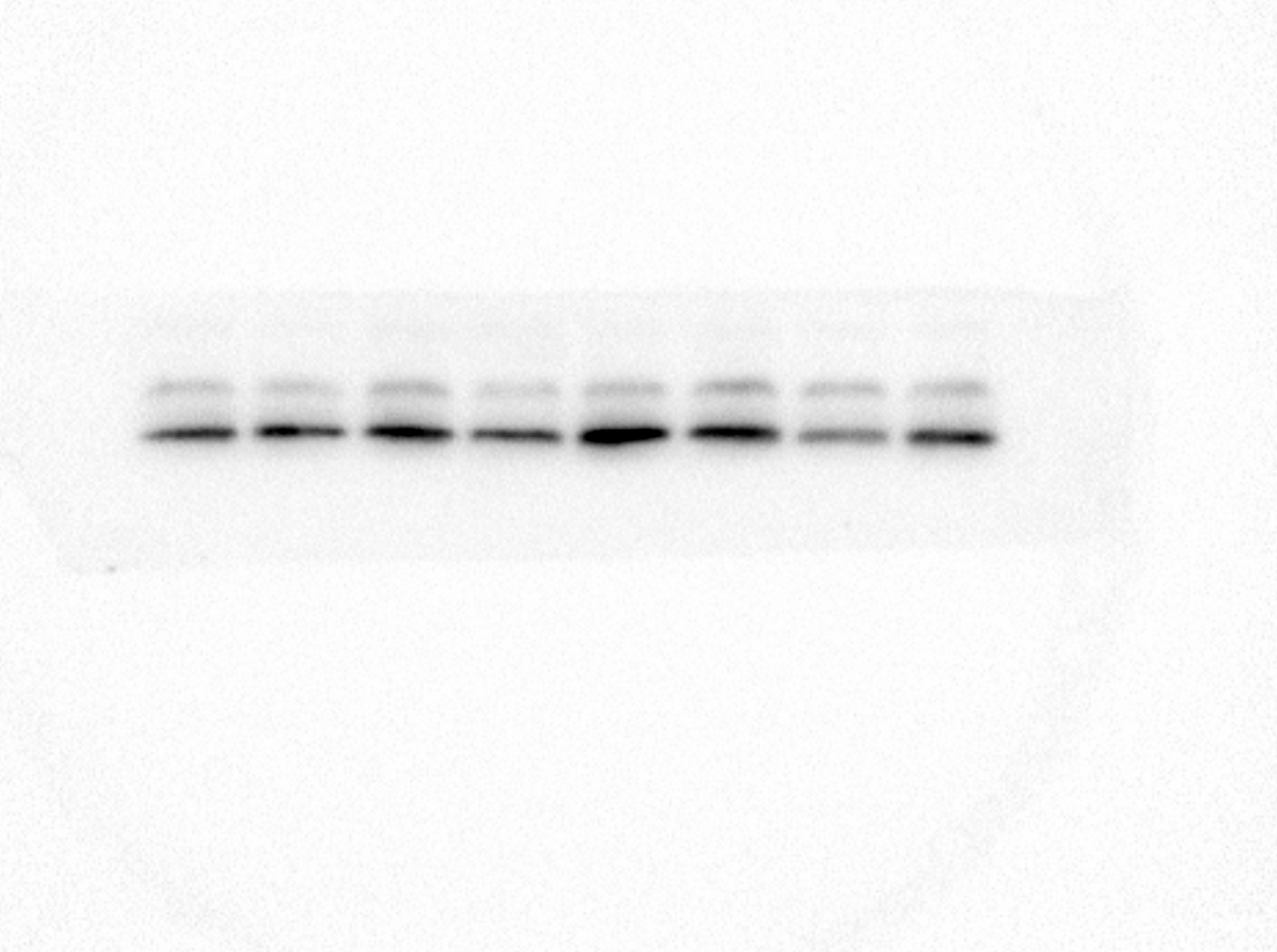

Supplement: Supplementary file 1 [file DataSheet_1.zip › supplementary materials (870174)/LC3 heart.tif]

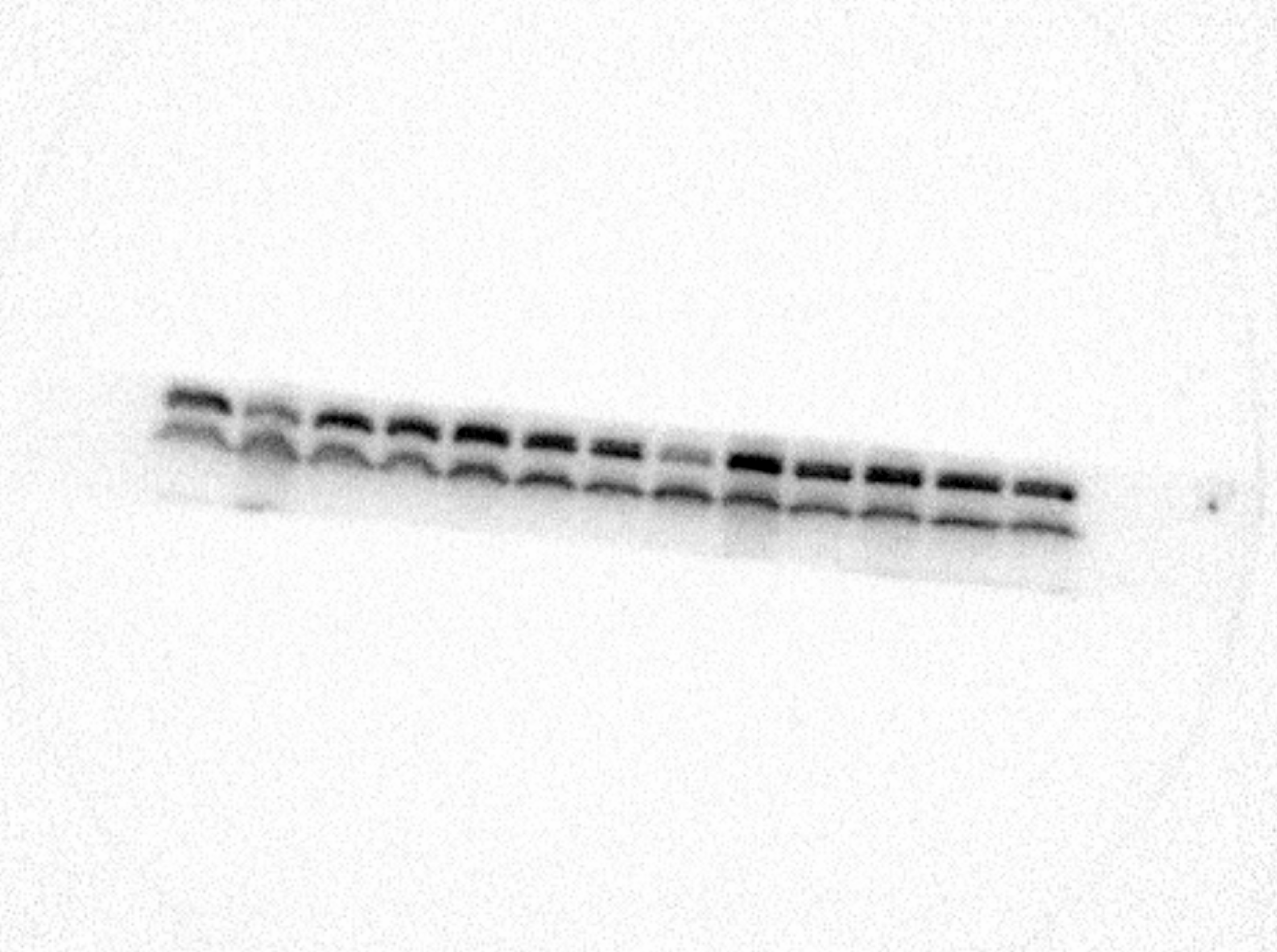

Supplement: Supplementary file 1 [file DataSheet_1.zip › supplementary materials (870174)/P62 brain.tif]

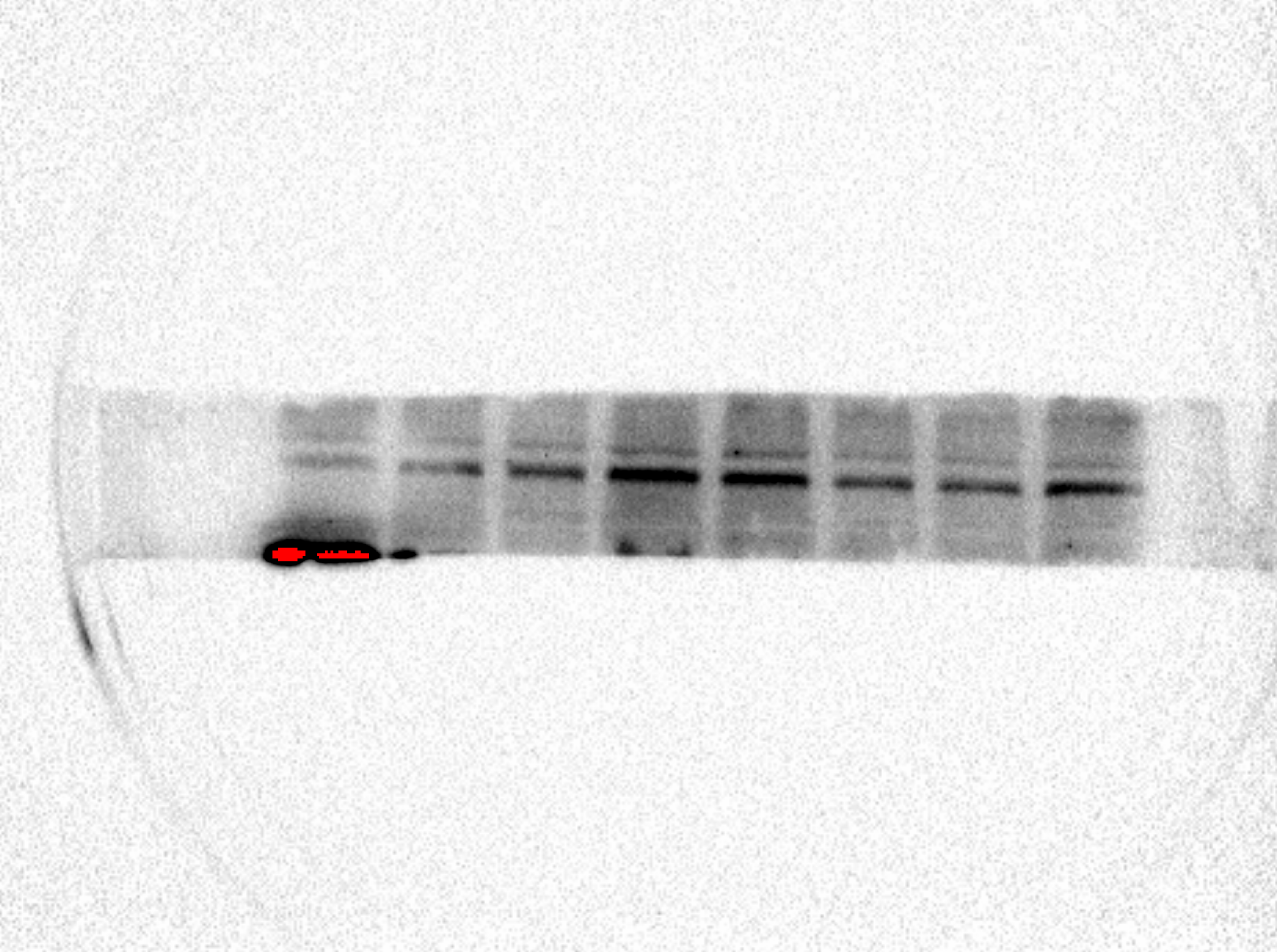

Supplement: Supplementary file 1 [file DataSheet_1.zip › supplementary materials (870174)/P62-Heart.tif]

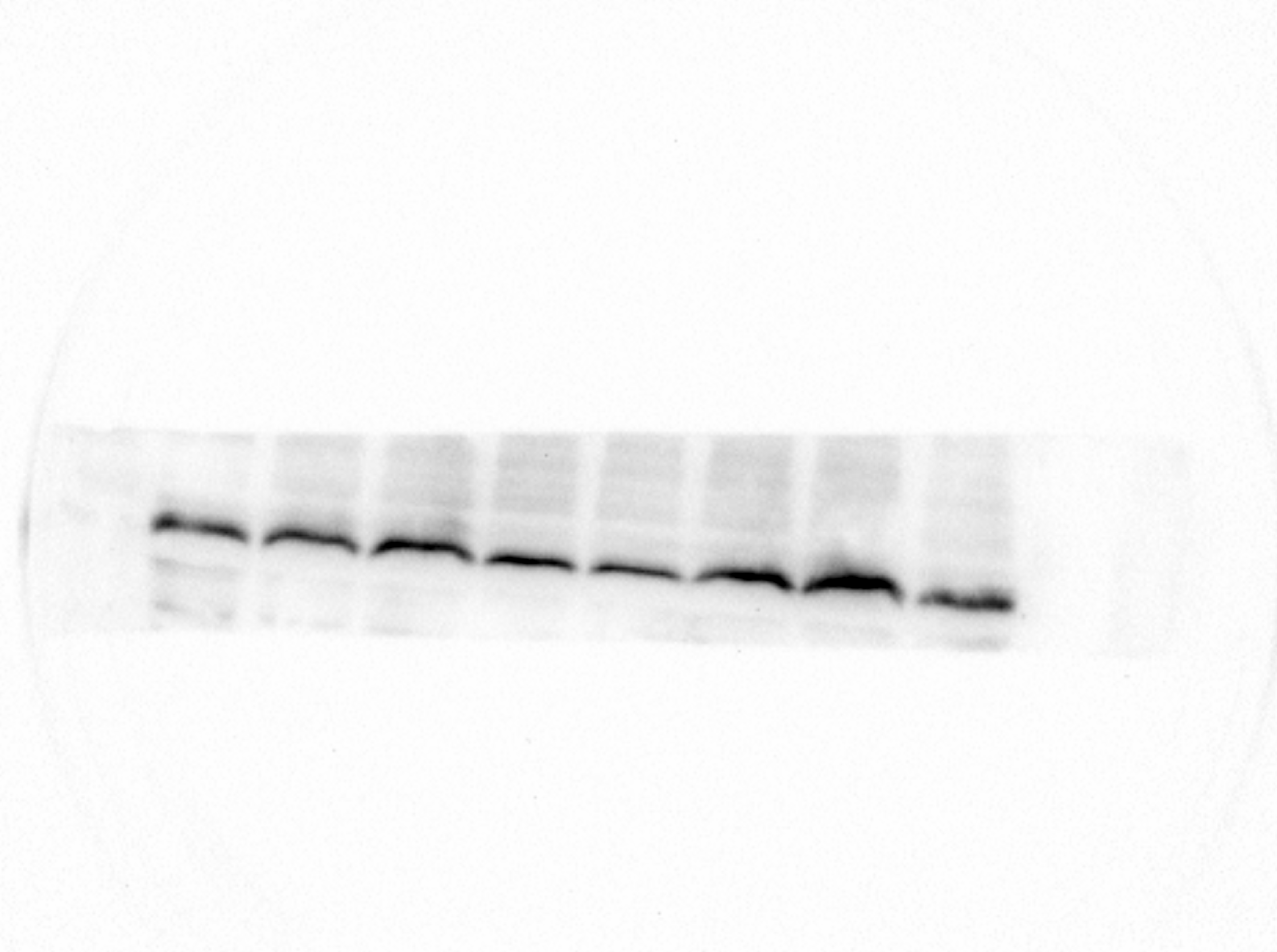

Supplement: Supplementary file 1 [file DataSheet_1.zip › supplementary materials (870174)/beclin-brain.tif]

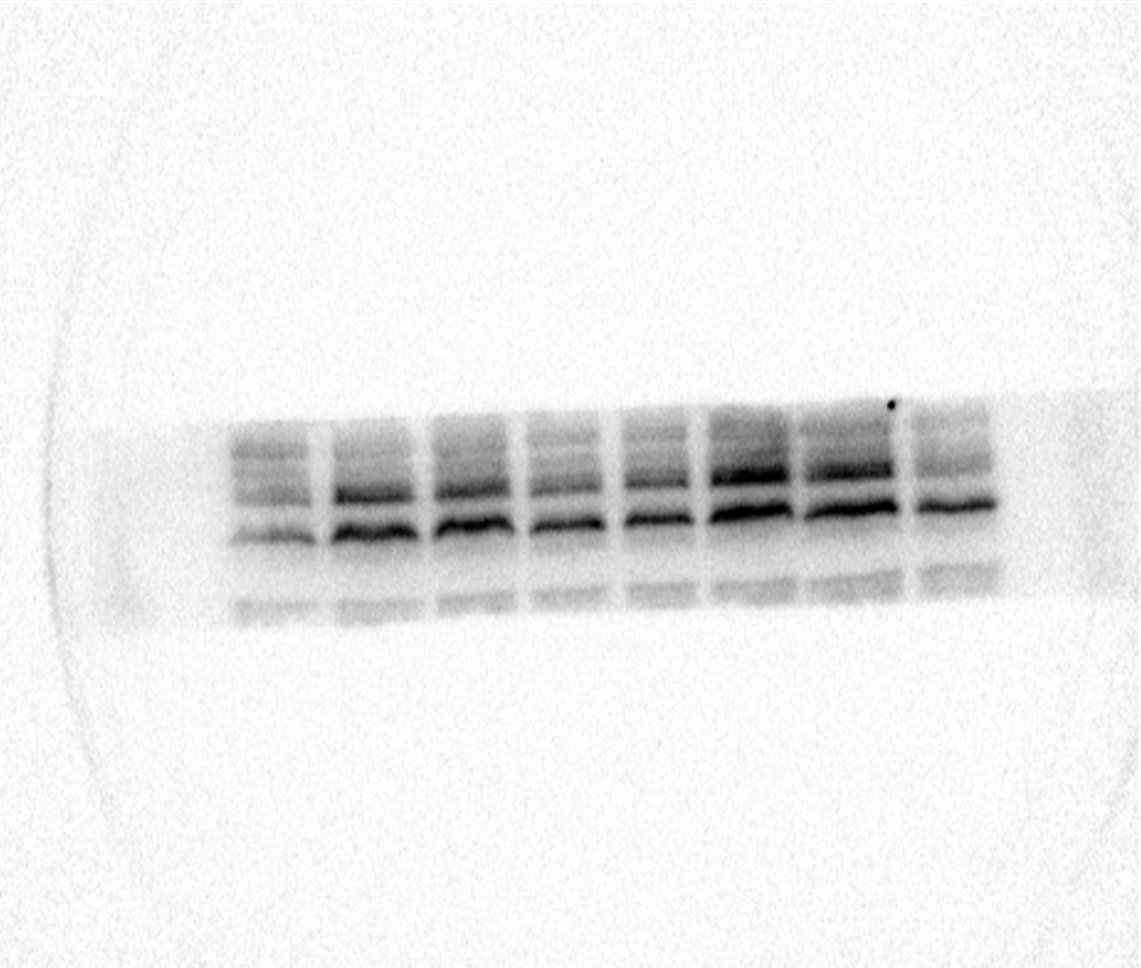

Supplement: Supplementary file 1 [file DataSheet_1.zip › supplementary materials (870174)/beclin-heart.tif]

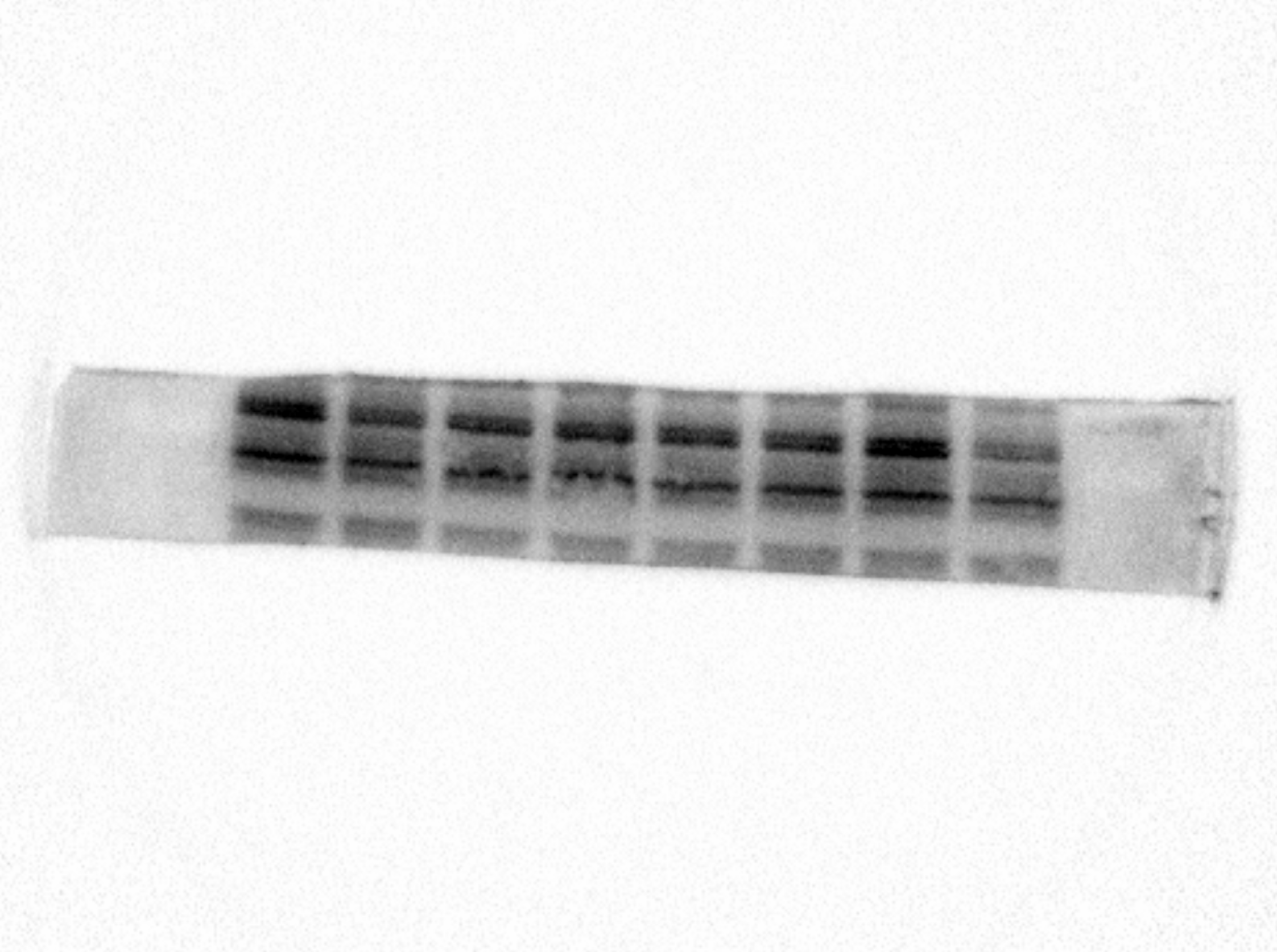

Supplement: Supplementary file 1 [file DataSheet_1.zip › supplementary materials (870174)/a┴7nAChR-brain.tif]

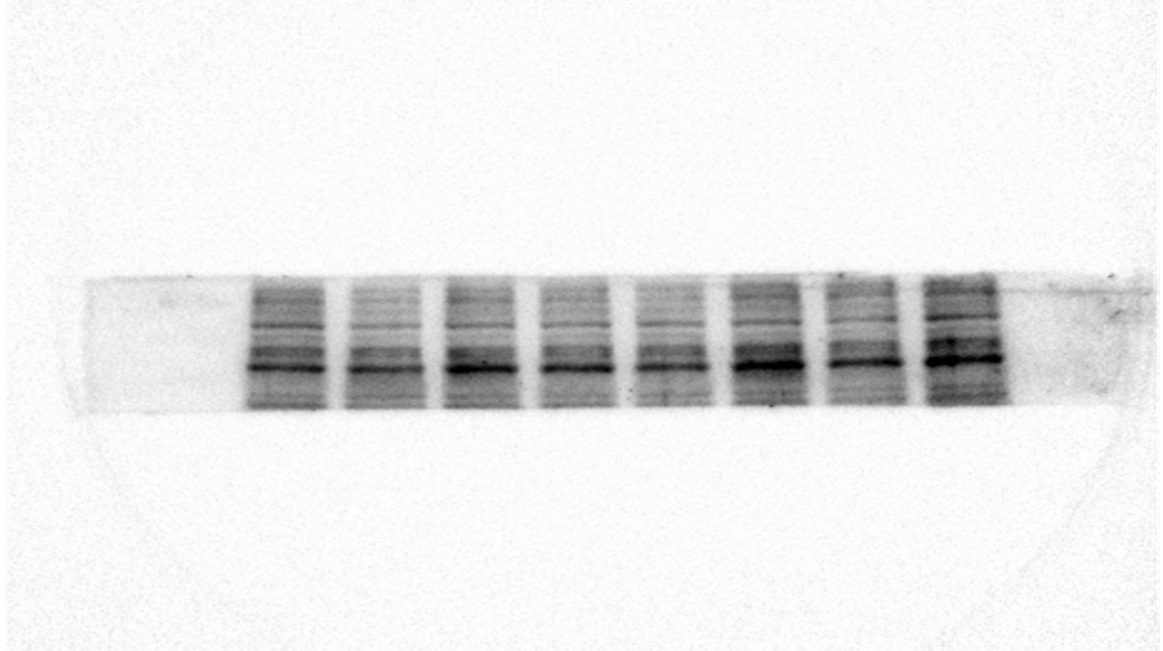

Supplement: Supplementary file 1 [file DataSheet_1.zip › supplementary materials (870174)/a┴7nAChR-heart.tif]
